# Promotion of emotional competence in nurses who cares people in end of life: a continuous improvement project for mental health nursing care

**DOI:** 10.1192/j.eurpsy.2025.876

**Published:** 2025-08-26

**Authors:** C. Laranjeira, A. Argüelles

**Affiliations:** 1 School of Health Sciences; 2 ciTechCare, Polytechnic Institute of Leiria, Leiria; 3 Medical-surgical specialty ward, Hospital Distrital de Santarém, Santarém, Portugal

## Abstract

**Introduction:**

The constant contact with death and dying processes increases the risk of mental suffering in nurses. The evidence suggests the need for training programs in the area of promoting emotional competence that allow the management of emotions and consequently the improvement of care for people at the end of life.

**Objectives:**

Capacitate a group of nurses to promote emotional competence towards end-of-life patients, through a specialized intervention in Mental Health and Psychiatric Nursing; and evaluate attitudes and coping with death and levels of emotional competence [and respective capabilities], pre and post-intervention.

**Methods:**

A continuous quality of care improvement project was carried out based on the Deming cycle, with pre and post intervention evaluation. The following instruments were applied: Veiga-Branco Emotional Competence Scale - Reduced (EVCE-reduced); Scale for Assessment of the Profile of Attitudes about Death; and, Coping with Death Scale. The training program consisted of seven in-person sessions, in groups, with approximation to the principles of psychoeducational intervention and based on the cognitive- behavioural sequence.

**Results:**

Eleven nurses participate in program. Most of respondents were female (90%) aged between 26 and 50 years (M=36±7.98). Regarding the coping with death scale, it was possible to observe in the factor “coping with one’s own death”, after application of the program, an overall average of 73.38 (SD=4.21), verifying statistically significant differences between the pre- and post-intervention (Z=1.963, p< 0.05). It was also possible to observe an increase in the global score of EVCE-reduced (M=170.01; SD=20.88), accompanied by a slight increase in capabilities: self-awareness (M=45.88; SD=4.64) and empathy (M=28.13; SD=3.44). Regarding the scale referring to the profile of attitudes towards death, there was an increase in the attitude of acceptance by approach (M=44.00; SD=12.22). On the contrary, there was a decrease in the fear attitude (M=24.63; SD=9.61), in the avoidance attitude (M=11.25; SD=4.49) and the attitude of acceptance as an escape (M=14.25; SD=4.50), compared to pre-intervention values.

**Conclusions:**

Our findings indicate that there is an integration of death as a natural event and an integral part of life. On the other hand, adequate management of fear of death and a non-avoidance attitude contribute to more compassionate care, and therefore more centered on the person at the end of life. Besides, this study allow us to confirm the importance of training programs in socio-emotional regulation, as they focus on the mobilization of intra and interpersonal resources, as well as the expressiveness of emotions in the face of one’s own death and the death of others, enabling the development of deeper levels of introspection.

**Disclosure of Interest:**

None Declared

